# Mediating Effect of Loneliness in the Relationship between Depressive Symptoms and Cognitive Frailty in Community-Dwelling Older Adults

**DOI:** 10.3390/brainsci12101341

**Published:** 2022-10-04

**Authors:** Ping Hou, Huiping Xue, Yu Zhang, Yujie Ping, Yijiang Zheng, Yan Wang, Zhenshuai Yao, Xinyi Xie, Hua Dai, Yongbing Liu

**Affiliations:** 1 School of Nursing and School of Public Health, Yangzhou University, Yangzhou 225000, China; 2 Nagano College of Nursing, Nagano 399-4117, Japan; 3 Affiliated Hospital of Nantong University, Nantong 226000, China; 4 Medical College, Yangzhou University, Yangzhou 225009, China

**Keywords:** cognitive frailty, depression symptoms, loneliness, older adults

## Abstract

Background: This study aims to explore the mediating role of loneliness between depressive symptoms and cognitive frailty among older adults in the community. Methods: A total of 527 community-dwelling older adults aged ≥ 60 years were included in this cross-sectional study. A five-item geriatric depression scale was used to assess depression symptoms. Then, an eight-item University of California at Los Angeles Loneliness Scale was used to assess loneliness. Moreover, the FRAIL scale and Mini-Mental State Examination were used to assess cognitive frailty. Furthermore, regression and bootstrap analyses were used to explore the mediating role of loneliness in depression symptoms and cognitive frailty. Results: Loneliness mediates the association between depression symptoms and cognitive frailty (95% CI = 0.164~0.615), and after adjusting for loneliness, the direct effect is no longer significant (95% CI = −0.113~1.318, *p* = 0.099). Conclusions: Results show that the effect of cognitive frailty is not depression symptoms but loneliness. All levels of society (the government, medical institutions, and communities) need to pay more attention to the mental health of the older adults, screen for loneliness, and take timely intervention and treatment measures. They should also build an age-friendly society and promote active aging.

## 1. Introduction

Cognitive frailty is a complex geriatric syndrome and is defined as simultaneous physical frailty and mild cognitive impairment (MCI), excluding Alzheimer’s disease and other types of dementia [1]. Cognitive frailty plays an important role in predicting adverse health outcomes, such as injuries, falls, and disability [2]. People with cognitive frailty have a significantly higher risk of dementia, death, and hospitalization than those without frailty or cognitive impairment [3,4]. The incidence of cognitive frailty increases year by year, and with the increase in age, its incidence is higher [5,6]. However, cognitive frailty may be reversed [7]. As the world’s population ages rapidly, it is urgent we pay more attention to cognitive frailty, accurately identify elderly people with cognitive frailty, explore its influencing factors, and prevent or delay it as soon as possible.

Depression symptoms are a common and complex mental disorder and have a high incidence rate worldwide. Several studies have shown that depression is associated with frailty [8,9]. They have similar pathophysiological mechanisms, including cerebrovascular disease, inflammation, and hypothalamic–pituitary–adrenal axis dysregulation [8]. A previous study found that older adults with depression have a high risk of cognitive frailty. The possibility of cognitive frailty in the elderly has increased 1.5 times because of depression [10]. Depression increases the risk of cognitive frailty in the frail elderly by 75% [11]. When untreated depression and frailty coexist, it increases the possibility of negative consequences for the elderly, and ultimately leads to cognitive frailty. However, the mechanism between depression and cognitive frailty is unclear.

Loneliness is a negative psychological feeling when a gap exists between the desired and the real social relationship (either quantitatively or qualitatively) [12]. As a public health problem, loneliness can increase the risk of cognitive impairment, dementia, and death [13,14]. The English longitudinal study of aging found that the risk of frailty increases with the severity of loneliness [15]. In Singaporean nursing homes, loneliness is significantly related to frailty. The prevalence of loneliness in the frail elderly is 1.37 times higher than that in the pre-frail [16]. Sha et al. indicated that in the frailty transition types, the more lonely the Chinese elderly are, the more they become frail in their later days. The higher the degree of loneliness, the stronger the possibility of deterioration of frailty [17]. Among the Chinese community-dwelling elderly, loneliness is a robust factor for cognitive impairment and frailty [18]. The results of a study involving middle-aged and elderly people in several low- and middle-income countries illustrated that loneliness is related to mild cognitive impairment [19]. The study suggested that reducing the incidence of loneliness in the population can maintain cognitive function and prevent the occurrence of dementia [19]. However, a study of older old people in Cambridge showed that loneliness does not have a long-term harmful effect on cognitive function [20]. The influence of loneliness on cognition is controversial, and little research has studied cognition and frailty as a whole. The effect of loneliness on cognition frailty needs to be explored urgently.

A systematic review of longitudinal studies reported that depression is a risk factor for loneliness [21]. Among older adults in Africa, depression can increase the risk of loneliness by 2.9 times [22]. Among the elderly in Turkey, a positive correlation exists between depression and loneliness, loneliness can predict depression, and depression can also affect loneliness [23]. Depression has a strong correlation with loneliness. With the incidence of depression, the prevalence of loneliness in the elderly has greatly increased. Reviewing previous studies, we found that depression symptoms are related to cognitive frailty, and depression can lead to loneliness. Loneliness may increase the risk of frailty and cognitive impairment. Therefore, we hypothesize that loneliness may play an important role in the relationship between depression and cognitive frailty. To the best of our knowledge, this hypothesis has not been studied in the elderly with cognitive frailty.

This study aims to explore the mediating role of loneliness between depression and cognitive frailty in older adults. First, this study investigates the association between loneliness, depression, and cognitive frailty in the elderly. Then, this study determines the mediating role of loneliness between depression and cognitive frailty. To our knowledge, this study is the first to explain the possible mechanism of how depression affects cognitive frailty. If clarified, this study can provide a new direction for preventing cognitive frailty.

## 2. Materials and Methods

### 2.1. Setting and Participants

This cross-sectional study was conducted from June 2020 to August 2020 in Jiangsu Province, mainland China. Participants were recruited in seven cities (Nanjing, Suzhou, Wuxi, Yangzhou, Changzhou, Nantong, and Lianyungang) of Jiangsu Province through the convenient sampling method.

The inclusion criteria were as follows: (1) community-dwelling older adults aged 60 and over; (2) willing and able to comply with the investigation. Then, the exclusion criteria were the following: (1) diagnosed with dementia; (2) severe cardiopulmonary insufficiency; (3) serious hearing or visual problems. A total of 550 questionnaires were distributed, and 527 valid questionnaires were recovered. The effective recovery rate was 95.82%.

Eight investigators with medical knowledge received a week-long training and were allowed to conduct the investigation only after passing the test to ensure the consistency of the method. After obtaining the written informed consent of the respondents, we collected data face-to-face in their households. The investigators explained the items that the respondents did not understand without prejudice and took back the questionnaire on site.

### 2.2. Measurement

#### 2.2.1. Cognitive Frailty

The assessment of cognitive frailty is divided into two steps. The first step is to screen for frailty, and the second step is to evaluate the cognitive state. When the older adults have both physical frailty (including pre-frailty and frailty) and cognitive impairment, it is recognized as cognitive frailty.

Frailty was assessed using the FRAIL scale [24], which consists of five questions: (1) Fatigue: have you often felt tired in the past 4 weeks? (2) Low resistance: is it difficult to climb one floor of stairs without auxiliary tools and the help of others? (3) Low ambulation: is it difficult to walk a block of about 500 m without auxiliary tools and the help of others? (4) Loss of body mass: has your body mass decreased by ≥5% in the past year? (5) Illness: did you have five or more diseases? The score range of this scale is 0–5 points. If the elderly answer “yes,” they will get “1” point, and if they answer “no,” they will get “0” point. The scores of 0, 1–2, and 3–5 points represent robust, pre-frailty, and frailty, respectively. Dong et al. [25] translated and applied this to Chinese community-dwelling elderly and found that the FRAIL scale has good validity and reliability. The FRAIL scale is convenient and time-saving, has a strong predictive validity, and is suitable for rapid screening in a large sample survey [26].

Cognitive function was assessed by Mini-Mental State Examination (MMSE), which was reliable and the most widely used [27]. MMSE can test time and place orientation, memory, attention and computation, naming, and others, with a total of 30 items; 1 score is given for each correct answer, and the total score is 30 points. The higher scores mean better cognitive function. According to the educational level of participants, illiteracy of ≤17 points, primary school of ≤20 points, junior high school of ≤22 points, and senior high school and above of ≤24 points are diagnosed as MCI [28].

#### 2.2.2. Depression Symptoms

Depression symptoms were measured by the five-item Geriatric Depression Scale (GDS-5) [29]. The scale consists of five items with a score range of 0–5. According to the participants’ answers, each item is given 0 or 1. The severity of depression symptoms was positively correlated with the score. The cut-off value of the scale is two points. People with a score less than two points indicate no depressive symptoms, and those with a score greater than or equal to two points indicate depressive symptoms. The GDS-5 is designed on the basis of the 15-item GDS and has been proven to be as accurate as GDS-15 in screening for depression in the elderly [30]. The GDS-5 significantly reduces evaluation time and has been used in the Chinese population [9].

#### 2.2.3. Loneliness

Loneliness was assessed using the eight-item University of California at Los Angeles (UCLA) Loneliness Scale (ULS-8) [31]. This scale is derived from the 20-item UCLA scale and has high validity and reliability [32]. ULS-8 contains eight self-report questions, including six forward questions and two reverse questions, which avoids the response bias of respondents. Each item was scored with a four-point Likert scale: 1 (never), 2 (rarely), 3 (sometimes), and 4 (always). The total score is the sum of the scores of each item, and the score ranges from 8 to 32. The higher the total score, the higher the degree of loneliness.

#### 2.2.4. Control Variables

Sociodemographic characteristics were gender, age, body mass index (BMI), education, marital status, household arrangement, and monthly income. BMI is calculated by dividing the weight (kg) by the square of height (m). Values of 18.5–23.9 were considered as normal BMI, <18.5 or >23.9 was considered as abnormal BMI [33]. Marital status is categorized as married and single. Single includes unmarried, widowed, and divorced.

Other covariates were cigarette smoking, alcohol drinking, polypharmacy, annual check-up, and sleep disorder. If the elderly smoke 100 or more cigarettes in their lives, they are considered smokers. If men drink more than 25 g a day, or women drink more than 15 g a day, they are considered drinking [34]. Polypharmacy was considered as taking 5 or more medicines every day [35]. Sleep disorder was measured using the Athens Insomnia Scale. This scale is an internationally recognized sleep quality self-assessment scale, including eight items, with a score range of 0–24 points. To distinguish the elderly with sleep disorders from the healthy population, a cut-off value of six points has been determined [36].

### 2.3. Statistical Analysis

All analyses were performed using SPSS version 23.0 (IBM Corp., Armonk, NY, USA). A *p* value of less than 0.05 is statistically significant. First, we used frequency (percentage) or mean (standard deviation) to describe the demographic characteristics of the sample. χ2 test, ANOVA, and student’s *t* test were used to compare the differences in cognitive frailty in each group. The mediating model proposed by MacKinnon and Dwyer [37] was used to test the mediating effect of loneliness on the relationship between depression and cognitive frailty: (1) the association between depression and loneliness was evaluated through linear regression analysis; (2) binary logistic regression was performed to explore the relationship between depression and cognitive frailty, multi-collinearity was checked by variance inflation factor (VIF), when VIF > 10, it indicates that there is serious multi-collinearity; (3) binary logistic regression was used to further test whether the relationship between depression and cognitive frailty weakened or became insignificant when loneliness was involved. All analyses were adjusted for gender, age, BMI, education, marital status, household arrangement, monthly income, cigarette smoking, alcohol drinking, polypharmacy, annual check-up, and sleep disorders. Finally, the bootstrap method was used to evaluate the total, indirect, and direct effects of the model [38]. When the 95% confidence interval (CI) of the indirect effect which is based on 5000 bootstrap samples does not contain 0, the indirect effect is significant.

### 2.4. Ethical Consideration

The study was conducted in accordance with the Declaration of Helsinki, and the protocol was approved by the Committee on the Ethics of the researcher’s University (No. YZUHL2020008). Each participant knew the purpose and process of this study and signed the informed consent form. All personal data were not disclosed.

## 3. Results

### 3.1. Characteristics of Participants

The study included 527 older adults whose characteristics are shown in Table 1. Among these participants, the incidence of depressive symptoms is 9.9%, the prevalence of cognitive frailty is 19.7%, and the loneliness score is 14.2 ± 4.3. Compared to the elderly without cognitive frailty, those with cognitive frailty live alone, smoke, polypharmacy, and have abnormal BMI, low monthly income, and sleep disorders.

### 3.2. Effect of Loneliness on the Association between Depression and Cognitive Frailty

Linear regression showed that after adjusting for control variables, depression is correlated with loneliness (*β* = 2.855, *p* < 0.001), that is, older adults with depression are more lonely, as shown in Table 2. There is no multi-collinearity in our data, all VIF values are less than 10. In Table 3, the model without mediators (loneliness) shows that depression is related to cognitive frailty (OR = 2.35, *p* = 0.018). When loneliness is included, depression is no longer significantly associated with cognitive frailty (OR 1.83, *p* = 0.099), and loneliness is still positively correlated with cognitive frailty (OR 1.13, *p* < 0.001).

**Figure 1:**
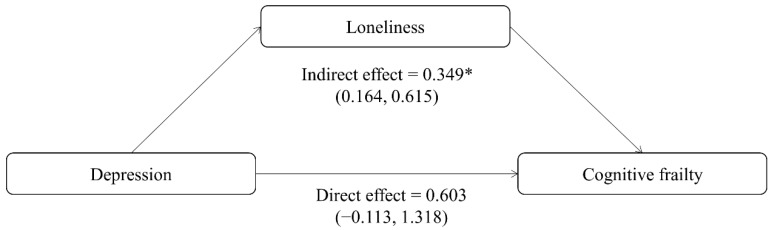
Mediating effects of loneliness on the association between depression symptoms and cognitive frailty in community-dwelling older adults. 95% confidence intervals are presented. Models control for gender, age, BMI, education, marital status, household arrangement, monthly income, cigarette smoking, alcohol drinking, polypharmacy, annual check-up, and sleep disorders. * *p* < 0.05.

The bootstrap test indicated that after adjusting for control variables, the direct effect of depression on cognitive frailty is 0.603 (95%CI = −0.113~1.318, *p* = 0.099). The indirect mediation effect of depression on cognitive frailty through loneliness is 0.349 (95% CI = 0.164~0.615), zero is outside the 95% CI, and the indirect effect is significant (Figure 1). Of the total effect of depression on cognitive frailty, 37% is explained by the mediating effect of loneliness. The direct effect of depression on cognitive frailty is no longer significant, indicating only indirect mediation.

## 4. Discussion

As far as we know, no research has tested the mediating role of loneliness between depression and cognitive frailty using the mediating model. Previous studies showed that depression is associated with cognitive frailty [10,39], but the potential mechanism has not been revealed. Our results demonstrated that older adults with depressive symptoms have a stronger sense of loneliness, which is related to their greater possibility of cognitive frailty.

In this study, the prevalence of cognitive frailty in Jiangsu province, mainland China, is 19.7%. It is higher than that of other studies in Taiwan (11.0%) and mainland China (6.2%) [40,41] and also higher than in Japan (9.8%) [4]. However, it is lower than the elderly in Hong Kong (35.7%) and Spain (21.8%) [10,42]. The difference in the prevalence of cognitive frailty may be caused by the different assessment tools. These studies used a variety of scales to assess cognitive function: for example, the national center for geriatrics and gerontology-functional assessment tool [4], MMSE [40,41], clinical dementia rating [10], and MoCA [42]. They used walking speed or muscle weakness [4], frailty phenotype [40,42], and FRAIL scale [10,41] to assess frailty. Moreover, in terms of the division of the frailty group, some articles included the participant in the pre-frailty/frailty stage [10,41,42], and some articles only included the elderly in the frailty stage [40]. In addition, different countries and regions have different lifestyles, economic levels, and medical technologies.

Depression is the most common mental health disorder among older adults. With the increasingly serious aging of the population, depression symptoms in older adults have attracted increasing attention. This study found that the prevalence of depressive symptoms in China (Jiangsu Province) is 9.9% and is close to Liu’s study (10.4% in Shandong Province, China) [9]. However, the prevalence is higher than in Lotfaliany’s study in China (1.5%) [43]. The differences may be because of the evaluation method of depression and reporting bias of the study population. Our depression prevalence is lower than a meta-analysis in mainland China (20%) [44]. The population included in that meta-analysis is diverse. Depression can be identified through a variety of means, and the areas involved are not limited to Jiangsu Province, resulting in the difference in the incidence of depression. The prevalence of depression in this study was lower than that in India (15.2%) and higher than that in South Africa (3.7%), which may be because of the differences in social culture, socio-economic status, and sample characteristics [43].

Our study revealed that depression is positively correlated with loneliness in the elderly. Compared with the elderly without depression, the elderly with depression were more likely to be lonely, which is consistent with the results of previous studies [21,22]. The results of a 14-year follow-up study to explore the temporal association between loneliness and depression showed that the temporal correlation between these two is bidirectional. In addition, the effect of depression on loneliness is stronger than that of loneliness on depression, and depression significantly predicts the incidence of loneliness at all observation points [45]. Sociodemographic factors may increase the incidence of loneliness and depression in the elderly. Taking Turkey as an example, when the labor force in the family, particularly women, go to work and no one takes care of the elderly at home, the incidence of loneliness and depression among these elderly people will rise [23]. Depression can destroy social relationships, impair interpersonal functioning, cause poor social engagement, and eventually lead to or aggravate loneliness [46,47]. Lack of social interaction can make older people feel lonely. Elderly people with depressive symptoms do not like to express their own needs, worry that they are not valued, take the initiative to reduce their contact with the outside world, and are even unwilling to engage in social communication. Depression prevents them from engaging in meaningful social relationships, resulting in loneliness.

Loneliness increases the risk of cognitive frailty. At present, direct research on loneliness and cognitive frailty is lacking. Some studies have demonstrated that loneliness is associated with cognitive impairment and frailty [18,19,48], reflecting the relationship between loneliness and cognitive frailty from the side. Loneliness has a high prevalence in people with low physical activity [49]. Loneliness may cause sarcopenia [15], which implies the loss of skeletal muscle mass, strength, and function. Sarcopenia can be caused by many factors, such as hormone regulation, myocyte changes, and inflammation. These pathological changes will reduce muscle strength, reduce physical activity, and eventually cause frailty [50]. Loneliness can lead to adverse health consequences, and loneliness is confirmed to be related to the coexistence of multiple diseases [51]. This finding indicates that the physical reserve capacity is insufficient, and the risk of frailty increases. Lonely people may be more likely to adopt an unhealthy lifestyle to deal with loneliness, which will be accompanied by a risk of cardiovascular disease, which is a recognized risk factor for cognitive decline [19]. People who experience loneliness have low social participation and insufficient sensory stimulation, which affects cognitive reserve and leads to low cognitive ability [52]. Loneliness triggers a neural response and affects the progress of neurodegenerative diseases. Loneliness and dementia are related to telomere shortening in leukocytes [53]. For people with normal cognition but who are lonely, their cortical amyloid burden increases, indicating that loneliness is a manifestation of preclinical Alzheimer’s disease [53].

A meta-analysis showed that whether in pre-frailty or in the frailty, frailty is closely related to the poor cognitive function [54]. The occurrence of cognitive frailty has brought great challenges to the healthcare system. Therefore, older people must adopt a positive lifestyle to delay its occurrence. The results of this study show that loneliness is a mediator between depression and cognitive frailty. Older adults with depression are often accompanied by loneliness, which increases the risk of cognitive frailty. The elderly with depressive symptoms had low social participation, escape, or lack of social interaction, resulting in loneliness. Lonely elderly people have insufficient muscle strength, less physical activity, poor social engagement, and lack of sensory stimulation. Coupled with unhealthy behaviors and other factors, this case can lead to the occurrence of low body function, cognitive decline, and frailty. Notably, depression can lead to loneliness, and loneliness is also a particularly important factor in the occurrence and development of depression [55]. Based on this, we also tried to analyze the mediating role of depressive symptoms in loneliness and cognitive frailty (Supplementary Material) and found a partial mediation effect of depressive symptoms on them. Depression and loneliness can affect each other and eventually cause cognitive frailty, which suggests that we must pay more attention to the psychological problems of the elderly, create more opportunities for communication and contact, and reduce depression and loneliness.

This study provides direction for the prevention and management of cognitive frailty caused by depression. First, loneliness plays an important role in the occurrence and development of cognitive frailty in the elderly with depression. However, the psychological state of the elderly is usually not as valued as physical diseases. Communities and medical institutions should pay more attention to, and screen, the psychological state of the elderly. Second, the state needs to strengthen the integration of the elderly into society and promote active aging. Many elderly people are forced to cut off social ties and cannot adapt to new social roles because of retirement. In addition, changes in their lifestyle can lead to depression and then loneliness. The government needs to strengthen efforts to set up more jobs for the elderly, enrich the courses at universities for the elderly, and hold more elderly activities in the community. Finally, children should pay more attention to their parents and encourage them to participate in activities so as to make them have something to do, learn, and enjoy in their old age, and reduce the incidence of cognitive frailty.

This study has the following limitations: first, as this is a cross-sectional study, the causality cannot be determined. Longitudinal studies are needed to verify causality in the future. Second, the variables in this study, such as “depression,” “loneliness,” and “frailty,” are subjectively reported by the participants themselves, and information bias may exist. Third, we just verified just one mediating variable, and the mediating effect of loneliness accounts for only 37% of the total effect. We need to continue exploring other potential variables related to cognitive frailty. Finally, the sample of this study is only from one province in China. Based on the differences in social culture, economy, and lifestyle, attention should be paid to the universality of the research results.

## 5. Conclusions

This study proved the relationship between loneliness and depression and cognitive frailty in the community-dwelling elderly for the first time. The effect of depression on cognitive frailty is mediated by loneliness. Cognitive frailty is a powerful risk factor leading to disability, dementia, and other adverse health consequences. Practical and effective measures need to be taken to prevent or delay its occurrence and development. Loneliness plays an important role between depression and cognitive frailty. Therefore, medical institutions and communities need to pay more attention to mental health, do a good job in screening loneliness in the elderly, and intervene in time. The government and relevant departments should improve policies and measures, support the reemployment of the elderly, increase the activity places of the elderly, create an elderly friendly social environment, and promote active aging.

## Figures and Tables

**Table 1:** Description and univariate analysis of cognitive frailty among community-dwelling older adults (*n* = 527).

Variable	Total	Cognitive Frailty		*X* ^2^ */t*	*p*-Value
		No	Yes		
Observations	527 (100.0)	423 (80.3)	104 (19.7)		
Depression symptoms				12.774	<0.001
Without depression symptoms	475 (90.1)	391 (82.3)	84 (17.7)		
With depression symptoms	52 (9.9)	32 (61.5)	20 (38.5)		
Loneliness	14.2 ± 4.3	13.7 ± 4.1	16.0 ± 4.4	−4.953	<0.001
Gender				0.012	0.914
Male	261 (49.5)	209 (80.1)	52 (19.9)		
Female	266 (50.5)	214 (80.5)	52 (19.5)		
Age				2.396	0.122
(60–69) years	279 (52.9)	231 (82.8)	48 (17.2)		
≥70 years	248 (47.1)	192 (77.4)	56 (22.6)		
BMI (kg/m^2^)				7.852	0.005
18.5–23.9	364 (69.1)	304 (83.5)	60 (16.5)		
<18.5 or >23.9	163 (30.9)	119 (73.0)	44 (27.0)		
Education				0.092	0.761
Primary school or below	316 (60.0)	255 (80.7)	61 (19.3)		
Junior high school and above	211 (40.0)	168 (79.6)	43 (20.4)		
Marital status				0.002	0.966
Married	386 (73.2)	310 (80.3)	76 (19.7)		
Single	141 (26.8)	113 (80.1)	28 (19.9)		
Household arrangement				20.337	<0.001
Empty-nest elderly	89 (16.9)	56 (62.9)	33 (37.1)		
Non-empty-nest elderly	438 (83.1)	367 (83.8)	71 (16.2)		
Monthly income (CNY)				21.332	<0.001
<2000	350 (66.4)	261 (74.6)	89 (25.4)		
≥2000	177 (33.6)	162 (91.5)	15 (8.5)		
Cigarette smoking				4.465	0.035
No	415 (78.7)	341 (82.2)	74 (17.8)		
Yes	112 (21.3)	82 (73.2)	30 (26.8)		
Alcohol drinking				1.822	0.177
No	373 (70.8)	305 (81.8)	68 (18.2)		
Yes	154 (29.2)	118 (76.6)	36 (23.4)		
Polypharmacy				8.875	0.003
No	349 (66.2)	293 (84.0)	56 (16.0)		
Yes	178 (33.8)	130 (73.0)	48 (27.0)		
Annual check-up				0.196	0.658
No	146 (27.7)	119 (81.5)	27 (18.5)		
Yes	381 (72.3)	304 (79.8)	77 (20.2)		
Sleep disorder				36.153	<0.001
No	300 (56.9)	268 (89.3)	32 (10.7)		
Yes	227 (43.1)	155 (68.3)	72 (31.7)		

BMI, body mass index; CNY, Chinese Yuan.

**Table 2:** Risk Factors for loneliness (*n* = 527).

Characteristics	*B*	SE	*β*	*p*-Value	VIF Value
Depression symptoms					
Without depression symptoms					
With depression symptoms	2.855	0.585	0.200	<0.001	1.032
Gender					
Male					
Female	−0.104	0.397	−0.012	0.794	1.338
Age					
(60–69) years					
≥70 years	−0.038	0.364	−0.004	0.917	1.119
BMI (kg/m^2^)					
18.5–23.9					
<18.5 or >23.9	1.025	0.380	0.111	0.007	1.047
Education					
Primary school or below					
Junior high school and above	−1.269	0.384	−0.146	0.001	1.203
Marital status					
Married					
Single	−0.803	0.459	−0.083	0.081	1.404
Household arrangement					
Empty-nest elderly					
Non-empty-nest elderly	−0.084	0.511	−0.007	0.869	1.245
Monthly income (CNY)					
<2000					
≥2000	−1.576	0.413	−0.175	<0.001	1.292
Cigarette smoking					
No					
Yes	0.924	0.479	0.089	0.054	1.304
Alcohol drinking					
No					
Yes	−0.080	0.444	−0.009	0.858	1.386
Polypharmacy					
No					
Yes	−0.541	0.370	−0.060	0.144	1.037
Annual check-up					
No					
Yes	1.399	0.418	0.147	0.001	1.185
Sleep disorder					
No					
Yes	−0.108	0.378	−0.013	0.775	1.187

BMI, body mass index; CNY, Chinese Yuan; VIF, variance inflation factor.

**Table 3:** The mediating effect of loneliness on the relationship between depressive symptoms and cognitive frailty (*n* = 527).

Characteristics	Model without Mediators			Model with Mediators		
	*OR*	95% CI	*p*-Value	*OR*	95% CI	*p*-Value
Depression symptoms						
Without depression symptoms						
With depression symptoms	2.35	1.16–4.78	0.018	1.83	0.89–3.74	0.099
Loneliness				1.13	1.06–1.20	<0.001
Gender						
Male						
Female	1.25	0.71–2.23	0.439	1.29	0.72–2.33	0.392
Age						
(60–69) years						
≥70 years	2.01	1.19–3.38	0.009	2.11	1.24–3.59	0.006
BMI (kg/m^2^)						
18.5–23.9						
<18.5 or >23.9	1.95	1.16–3.28	0.012	1.76	1.03–2.98	0.037
Education						
Primary school or below						
Junior high school and above	1.72	1.00–2.97	0.051	2.16	1.23–3.82	0.008
Marital status						
Married						
Single	0.44	0.23–0.85	0.013	0.47	0.24–0.89	0.021
Household arrangement						
Empty-nest elderly						
Non-empty-nest elderly	0.29	0.15–0.54	<0.001	0.30	0.15–0.57	<0.001
Monthly income (CNY)						
<2000						
≥2000	0.31	0.16–0.61	0.001	0.36	0.18–0.70	0.003
Cigarette smoking						
No						
Yes	1.67	0.88–3.16	0.114	1.54	0.81–2.93	0.192
Alcohol drinking						
No						
Yes	1.15	0.62–2.16	0.657	1.21	0.64–2.27	0.564
Polypharmacy						
No						
Yes	1.64	1.00–2.71	0.052	1.83	1.09–3.05	0.022
Annual check-up						
No						
Yes	0.92	0.52–1.64	0.784	0.78	0.43–1.40	0.398
Sleep disorder						
No						
Yes	4.75	2.76–8.18	<0.001	5.34	3.04–9.37	<0.001

BMI, body mass index; CNY, Chinese Yuan.

## Data Availability

The datasets used and/or analyzed during the current study are available from the corresponding author on reasonable request.

## Supplementary Materials

The following supporting information can be downloaded at: www.mdpi.com/article/10.3390/brainsci12101341/s1, Table S1: Risk Factors for depression symptoms; Table S2: The mediating effect of depressive symptoms on the relationship between loneliness and cognitive frailty; Figure S1: Mediating effects of depression symptoms on the association between loneliness and cognitive frailty in community-dwelling older adults.
