# Supplementary material for: Mediating Effect of Loneliness in the Relationship between Depressive Symptoms and Cognitive Frailty in Community-Dwelling Older Adults
Source: Brain Sci. 2022 Oct 4;12(10):1341. doi: 10.3390/brainsci12101341 (PMC9599707; doi:10.3390/brainsci12101341)
Supplement: Supplementary file 1 [file brainsci-12-01341-s001.zip › brainsci-1887886-supplementary.pdf]

Supplementary Material

Mediating effect of depressive symptoms in the relationship between loneliness and cognitive frailty in community-dwelling older adults

Statistical Analysis

All analyses were performed using SPSS version 23.0 (IBM Corp., Armonk, NY, USA). A *P* value of less than 0.05 is statistically significant. The mediating model proposed by MacKinnon and Dwyer was used to test the mediating effect of depression on the relationship between loneliness and cognitive frailty: (1) the association between loneliness and depression was evaluated through linear regression analysis, (2) binary logistic regression was performed to explore the relationship between loneliness and cognitive frailty，multi-collinearity was checked by variance inflation factor (VIF), when VIF > 10, it indicates that there is serious multi-collinearity; (3) binary logistic regression was used to further test whether the relationship between loneliness and cognitive frailty weakened or became insignificant when depression was involved. All analyses were adjusted for gender, age, BMI, education, marital status, household arrangement, monthly income, cigarette smoking, alcohol drinking, polypharmacy, annual check-up, and sleep disorders. Finally, the bootstrap method was used to evaluate the total, indirect, and direct effects of the model. When the 95% confidence interval (CI) of the indirect effect which is based on 5000 bootstrap samples does not contain 0, the indirect effect is significant.

Table S1. Risk Factors for depression symptoms (N=527)

Characteristics	*B*	SE	*β*	*P*-value	VIF value
Loneliness	0.033	0.007	0.206	<0.001	1.146

VIF, variance inflation factor

Models control for gender, age, BMI, education, marital status, household arrangement, monthly income, cigarette smoking, alcohol drinking, polypharmacy, annual check-up, and sleep disorders.

Table S2. The mediating effect of depressive symptoms on the relationship between loneliness and cognitive frailty (N=527)

Characteristics	Model without mediators			Model with mediators		
	OR	95% CI	*P*-value	*OR*	95% CI	*P*-value
Loneliness	1.138	1.071-1.209	<0.001	1.128	1.060-1.200	<0.001
Depression symptoms				1.453	1.066-1.982	0.018

Models control for gender, age, BMI, education, marital status, household arrangement, monthly income, cigarette smoking, alcohol drinking, polypharmacy, annual check-up, and sleep disorders.

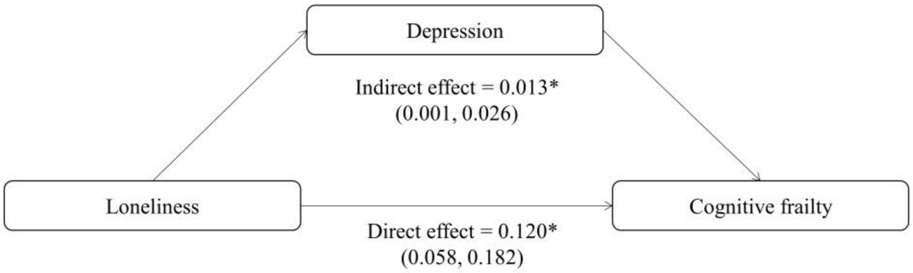

Figure S1. Mediating effects of depression symptoms on the association between loneliness and cognitive frailty in community-dwelling older adults.
95% confidence intervals are presented. Models control for gender, age, BMI, education, marital status, household arrangement, monthly income, cigarette smoking, alcohol drinking, polypharmacy, annual check-up, and sleep disorders. *P<0.05.
